# An all-inclusive approach: A universal protocol for the successful amplification of four genetic loci of all Onscidea

**DOI:** 10.1016/j.mex.2022.101762

**Published:** 2022-06-18

**Authors:** Andreas C. Dimitriou, Spyros Sfenthourakis

**Affiliations:** Department of Biological Sciences, University of Cyprus, Panepistimiou Ave. 1, 2109, Aglantzia, Nicosia, Cyprus

**Keywords:** PCR, Sanger sequencing, COI, 16S, 28S, NaK

## Abstract

Accounting more than 3,700 described species, Oniscidea is the largest and at the same time the only terrestrial isopod suborder inhabiting almost all terrestrial biomes. Despite the great effort dedicated on describing taxonomic diversity of Oniscidea, mainly employing morphology, there is still a considerable number of species/genera of uncertain generic/familiar assignment. Based on different morphological characters, alternative evolutionary relationships have been proposed to describe the diversity of Oniscidea at different phylogenetic levels. Accumulating morphological and genetic data are repeatedly challenging the monophyly of established taxa, undermining the validity of several morphological characters traditionally used in terrestrial isopod taxonomy, leading to often revisions of the current taxonomy of the Oniscidea . The use of genetic data facilitates the efforts to reconstruct the complex evolutionary history of the focal group by providing important data for the identification, delimitation, and description of species. The proposed protocol with universal PCR conditions and primers was used to successfully amplify COI, 16S, 28S and NAK loci in diverse Oniscidea taxa. The application of this protocol is anticipated to facilitate the generation of new genetic data and hence promote scientific research in Isopoda taxonomy, evolution, ecology, and other related fields.

## Graphical abstract

**Figure:**
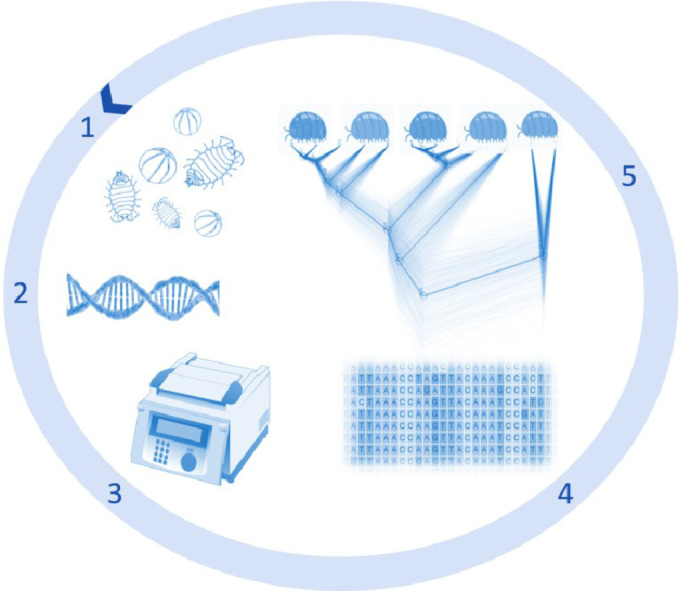

**SPECIFICATIONS TABLE**

**Table:** 

Subject Area;	Biochemistry, Genetics and Molecular Biology
More specific subject area;	PCR, Gene amplification
Protocol name;	A universal protocol for the successful amplification of four genetic loci of all Oniscidea
Reagents/tools;	PCR machine Primer A Primer B dNTPs MgCl_2_ Taq polymerase Taq buffer
Experimental design;	Herein we propose a universal easy and cost-effective way to produce genetic data for all Oniscidea. Multiple combinations and newly designed primers were tested under the same amplification conditions at a wide variety of taxa. Using the same primers for all targeted taxa the same gene fragments are targeted and hence produced data could be used to generate new or enrich existing datasets.
Trial registration;	*N/A*
Ethics;	*N/A*
Value of the Protocol;	• Universal for all Oniscidea • Simple and easy to follow protocol • Sequencing of the most popular genetic markers for taxonomic and phylogenetic studies • Cost effective

**Description of protocol:** Touchdown PCR was initially developed as a modification to traditional PCR aiming to increase the specificity and sensitivity of amplification [1], [2], [3]. The sensitivity of the proposed protocol was tested for four commonly targeted genetic loci for many taxonomically diverse taxa. The proposed protocol could be used to generate mitochondrial (16S, COI) and nuclear (28S, NaK) data.

**Required reagents and equipment**

PCR thermal cycler

PCR tubes

Sterile 1.5 mL Eppendorf style microcentrifuge tubes

Benchtop microcentrifuge

Adjustable micropipettes (0.1–1,000 mL)

Micropipette tips (10–1,000 mL) dNTPs

MgCl_2_

Taq polymerase

Taq Buffer

Primers (Table 1)

**Table 1:** Proposed primers for each locus.

Gene	Primer name	5′ -Sequence -3′	Product size	Reference
COI	LCO1490	GGT CAA CAA ATC ATA AAG ATA TTG G	∼560	[4]
	IsoCoiRint	GCY CCY GCY AAW ACA GGK ARD GA		[5]
16S	16sTRF	CTG ACT GTG CTA AGG TAG CA	∼375bp	This study
	16sTRH	CGG TYT GAA CTC AGA TCA YGT GA		
28S	28S a	GAC CCG TCT TGA AAC ACG GA	350bp - 800bp	[6]
	28S b	TCG GAA GGA ACC AGC TAC TA		
NaK	NakForb	ATG ACA GTT GCT CAT ATG TGG TT	∼600bp	[7] [8]
	Nak638R	GGD RGR TCR ATC ATD GAC AT		

## Method details

The proposed final reaction volume is 20 μL and consists of 0.1 µL of Taq DNA Polymerase (5U/μL), 2.4 μL of 25 mM MgCl_2_, 1X of Taq buffer A, 0.6 μL of 10 mM dNTPs, 0.6 μL of each primer (10 µM; Table 1) and >2 ng of DNA template in case of COI and 16s or >10ng in case of 28s and NAK. In any case the use of more than 100ng as DNA template should be avoided. The proposed protocol works effectively with samples of high DNA purity i.e..  A260/A280 rates over 1.5.

Thermocycling conditions for all four proposed genes should be as follows:

a) 94°C 10 min initial denaturation 1 cycle
b) 94°C 60s, 55°C 60s, 72°C 60s for 5 cycles
c) 94°C 60s, 50°C 60s, 72°C 60s for 5 cycles
d) 94°C 60s, 47°C 60s, 72°C 60s for 32 cycles
e) 94°C 60s, 42°C 60s, 72°C 60s for 7 cycles
f) 72°C 10 min final extension 1 cycle
g) *Hold 10*°C

The proposed protocol works efficiently even at very low DNA concentrations (<2ng), ill preserved or very old specimens. If aspecific products are amplified under these conditions an alternative, stricter thermocycling profile should be followed:

a) 94°C 10 min initial denaturation 1 cycle
b) 94°C 60s, 55°C 60s, 72°C 60s for 10 cycles
c) 94°C 60s, 50°C 60s, 72°C 60s for 10 cycles
d) 94°C 60s, 47°C 60s, 72°C 60s for 30 cycles
e) 72°C 10 min final extension 1 cycle
f) *Hold 10*°C

PCR products may be visualized using a common agarose gel electrophoresis. Successfully amplified samples should be purified using any commercial purification protocol following the manufacturer's instructions before proceeding with sequencing. Cycle sequencing can be performed with the same primers used for the initial PCR reaction.

## Validation

The effectiveness of the proposed protocol was tested for all available specimens, representing 142 species in 97 genera and 22 families, representing a diverse cross-section of the Oniscidea. Beyond terrestrial isopods, the same protocol was also successfully applied for representatives of the isopod orders Valvifera, Sphaeromatidea and Asellota.

Generated data were deposited in NCBI GenBank (Table 2). A limited number of sequences produced from our group within the framework of other projects, using a very similar protocol, were already published within the framework of previews studies and their corresponding accession numbers are given in Table 1. [8,9]. Although the new protocol using proposed thermocycling profile and primers were successfully tested in these cases too.

**Table 2:** Species, locality of origin and GenBank accession numbers of individuals used for the protocol validation. Isolate codes with stars (*) indicate that they were previously published [8,9].

Isolate code	Species	16S	COI	28S	NaK	Origin
Oniscidea						
Crinocheta						
Agnaridae						
55z*	*Agnara madagascariensis* (Budde-Lund, 1885)	—	—	MG888003	MG887924	U.A.Emirates
77Y	*Desertoniscus zaitsevi* Gongalsky, 2017	ON219990	ON212532	—	—	Bolshoy Tsaryn (Russia)
183z		ON219989	ON212533	ON312023	—	Bolshoy Tsaryn (Russia)
184z		ON219988	ON212534	ON312022	—	Bolshoy Tsaryn (Russia)
35h	*Hemilepistus elongatus* Budde-Lund, 1885	ON220006	ON212527	—	—	Kerman Anar (Iran)
23h		ON220001	ON212528	ON312034	—	Shirvan (Iran)
34h		ON220005	ON212529	—	—	Semnan Douzahir (Iran)
31h		ON220002	ON212525	—	—	Marand (Iran)
32h		ON220003	ON212526	—	—	Shahrood (Iran)
33h		ON220004	ON212497	—	—	Shahrood (Iran)
24h*	*Hemilepistus reaumurii* (Milne-Edwards, 1840)	ON220016	ON212514	MN174828	MN234258	Medenine (Tunisia)
27h		ON220019	ON212516	—	—	Libya
29h		ON220021	ON212519	—	—	Jordan
30h		ON220022	ON212520	—	—	Syria
25h		ON220017	ON212515	—	—	Tunisia
26h		ON220018	ON212517	—	—	Jordan
28h		ON220020	ON212518	ON312036	—	Syria
2h*	*Hemilepistus schirazi* Lincoln, 1970	—	—	MG888012	MG887927	Isfahan (Iran)
6h		ON220026	ON212493	ON312031	—	Isfahan (Iran)
8h		ON220027	ON212494	ON312032	—	Talkhuncheh (Iran)
3h		ON220023	ON212496	ON312028	ON227363	Zahedshahr Nasirabad (Iran)
4h		ON220024	ON212492	ON312029	—	Neyriz (Iran)
5h		ON220025	ON212495	ON312030	ON227366	Arsanjan (Iran)
36h	*Hemilepistus taftanicus* Kashani, Sari & Hosseini, 2010	ON220028	ON212498	—	—	Kerman (Iran)
37h		ON220029	ON212499	—	—	Khash sistan (Iran)
15h	*Hemilepistus aphganicus* Borutzky, 1958	ON219997	ON212500	—	—	Mashhad (Iran)
16h		ON219998	ON212501	ON312033	ON227368	Mashhad (Iran)
17h		ON219999	ON212502	—	ON227367	Razavi Khorasan Saraks (Iran)
18h		ON220000	ON212503	—	ON227370	Torbate-Jam (Iran)
7h	*Hemilepistus klugii* (Brandt, 1833)	ON220014	—	ON312027	ON227362	Meymeh (Iran)
9h		ON220015	—	—	ON227365	Hosnijeh (Iran)
19h		ON220012	ON212511	—	—	Qazvin,Bouin-Zahra (Iran)
1h*		MG887951	MG887938	MG888011	MG887926	Isfahan (Iran)
7h		—	ON212504	—	—	Meymeh (Iran)
9h		—	ON212505	—	—	Hasanijeh (Iran)
10h		ON220007	ON212506	ON312035	ON227364	Isfahan kashan Borzak (Iran)
11h		ON220008	ON212509	—	ON227369	Zavieh (Iran)
12h		ON220009	ON212508	—	—	Komijan (Iran)
13h		ON220010	ON212507	—	—	Mahallat (Iran)
14h		ON220011	ON212512	—	—	Mahallat (Iran)
64z	*Mongoloniscus sinensis* (Dollfus, 1901)	—	—	ON312044	—	Koko Nor Lake (China)
3z	*Orthometopon phaleronense* (Verhoeff, 1901)	ON220049	—	ON312049	—	Pertouli (Greece)
142z*	*Protracheoniscus* af. *fossuliger* Verhoeff, 1901	—	—	MN174817	MN234281	Greece
181z	*Protracheoniscus major* (Dollfus, 1903)	ON220053	ON212531	ON312053	—	Bolshoy Tsaryn (Russia)
182z		ON220054	ON212530	ON312054	ON227371	Bolshoy Tsaryn (Russia)
Alloniscidae						
63z	*Alloniscus oahuensis* Budde-Lund, 1885	ON219960	—	—	—	Bali (Indonesia)
Armadillidae						
47cy*	*Armadillo officinalis* Duméril, 1816	ON219965	—	MN174812	MN234252	Larnaca Salt Lake (Cyprus)
48cy		ON219966	—	ON312006	ON227376	Agios Theodoros (Cyprus)
51cy		—	—	ON312007	—	Agia Varvara (Cyprus)
56cy		—	—	ON312008	—	Geri (Cyprus)
12z	*Buddelundia bipartita* (Budde-Lund, 1912)	ON219970	—	—	—	Austalia
59z	*Cubaris nepalensis* (Vandel, 1973)	—	—	ON312019	—	Ilam (Nepal)
96z	*Sphaerillodillo pubescens* (Budde-Lund, 1885)	—	—	ON312024	—	Eastern Cape Transkei (South Africa)
98z	*Laureola hiatus* Barnard, 1960			ON312037	—	Haenertsburg (South Africa)
78z	*Lobodillo hebridarum* (Verhoeff, 1926)	ON220041	—	—	—	Vanuatu islands
99z	*Pseudodiploexochus* sp*.*	—	—	ON312055	—	Moramanga (Madagascar)
100z	*Pseudolaureola hystrix* (Barnard, 1958)	—	—	ON312056	—	Moramanga (Madagascar)
83z	*Venezillo* sp.	—	—	ON312087	—	Caldera de Bandama (Canary Islands)
Armadillidiidae						
52cy	*Armadillidium fallax* Brandt, 1833	ON219962	ON212577	—	—	Cape Greco (Cyprus)
87cy		ON219963	—	—	—	Akrotiri (Cyprus)
34z	*Armadillidium vulgare* (Latreille, 1804)	—	ON212545	—	—	Limassol (Cyprus)
55cy*		—	ON212537	—	—	Limnatis (Cyprus)
66cy		ON219964	—	ON312005	—	Arakapas (Cyprus)
62T	*Armadillidium cavernarum* Vandel, 1958	—	—	—	—	Cave Katofygi, Crete (Greece)
151z*	*Cyphodillidium absoloni* (Strouhal, 1934)	ON219983	ON212539	MN174814	MN234276	Saplunara (Croatia)
156z	*Echinarmadillidium fruxgalii* (Verhoeff, 1900)	ON219993	—	—	—	Baška voda (Croatia)
157z		ON219994	ON212538	—	—	Lumbarda (Croatia)
100cy	*Schizidium fissum* (Budde-Lund, 1885)	—	—	ON312057	—	Pitargou (Cyprus)
100cy		ON220056	ON212540	MG888005	—	Pitargou (Cyprus)
75T	*Troglarmadillidium halophilum* Sfenthourakis, 1993	—	ON212618	—	—	Antikythira (Greece)
179z*	*Typhlarmadillidium* sp.	ON220106	—	MN174815	MN234273	Gromača (Croatia)
180z		ON220107	—	—	—	Krčevine (Bosnia and Herzegovina)
79z	*Australiodillo bifrons* (Budde-Lund, 1885)	ON219967	—	—	—	Alligator Creek (Australia)
Balloniscidae						
49z	*Balloniscus sellowi* (Brandt, 1833)	—	—	ON312009	—	Montevideo (Uruguay)
Cylisticidae						
143z*	*Cylisticus convexus* (De Geer, 1778)	—	—	MN174813	MN234280	Budapest (Hungary)
Detonidae						
72z	*Deto marina* (Chilton, 1885)	ON219991	—	—	—	Mount Agnew (Tasmania)
Eubelidae						
11z	*Dioscoridillo melanoleucos* Ferrara & Taiti, 1996	ON219992	—	—	—	Socotra (Yemen)
86z	*Microcercus* sp.	—	—	ON312041	—	Lake Bogoria (Kenya)
57z	*Periscyphis omanensis* Taiti & Ferrara, 1991	—	—	—	ON227374	Dhofar (Oman)
Halophilosciidae						
68cy	*Halophiloscia* sp.	ON219996	—	—	—	Fontana Amorosa (Cyprus)
99cy		—	—	ON312025	—	Zygi (Cyprus)
120z	*Halophiloscia zosterae* (Verhoeff, 1928)	—	—	ON312026	—	Tinos (Greece)
44z	*Littorophiloscia tropicalis* Taiti & Ferrara, 1986	—	—	ON312039	—	Hispaniola island
Oniscidae						
141z	*Oniscus asellus* Linnaeus, 1758	—	—	ON312047	—	Budapest (Hungary)
169z	*Oroniscus dalmaticus* Strouhal, 1937	ON220046	ON212548	—	—	Jujnovići (Croatia)
170z		ON220047	—	—	—	Antunovići (Croatia)
171*		—	—	ON312048	—	Golubinjak (Croatia)
171*		ON220048	ON212547	MN174816	MN234274	Golubinjak (Croatia)
39z	*Phalloniscus mateui* Vandel, 1953	—	—	ON312050	—	Segovia (Spain)
203z	*Rodoniscus anophthalmus* Arcangeli, 1934	ON220055	ON212544	—	ON227373	Iraklia island (Greece)
Philosciidae						
28z	*Benthana trinodulata* Araujo & Lopes, 2003	—	—	ON312010	—	Bahia (Brazil)
35T	*Chaetophiloscia cellaria* (Dollfus, 1884)	ON219979	—	—	—	Nychteridospilios, Crete (Greece)
19cy	*Chaetophiloscia elongata* (Dollfus, 1884)	—	—	ON312015	—	Arakapas (Cyprus)
8z*		—	—	—	MG887929	Sardinia
105cy	*Chaetophiloscia lagoi* (Arcangeli, 1934)	—	ON212543	ON312016	—	Larnaca (Cyprus)
93z	*Congophiloscia longiantennata* Schmalfuss & Ferrara, 1978	—	ON212578		ON227375	Kribi (Cameroon)
40z	*Ctenoscia minima* (Dollfus, 1892)	—	—	ON312018	—	Mallorca
162z	*Lepidoniscus minutus* (C. Koch, 1838)	ON220032	ON212559	—	—	Zaprešić (Croatia)
163z		ON220033	—	—	—	Zaprešić (Croatia)
5z	*Philoscia affinis* Verhoeff, 1908	ON220050	—	—	—	Pedra Longa (Sardinia)
27z	*Androdeloscia lejeunei* (Lemos de Castro & Souza, 1986)	—	—	ON312004	—	Brazil
Platyarthridae						
32z*	*Platyarthrus schoblii* Budde-Lund, 1885	—	—	MN174833	MN234254	Pitargou (Cyprus)
140z*	*Trichorhina heterophthalma* Lemos de Castro, 1964	—	—	MN174845	MN234282	The Netherlands (greenhouse)
Porcellionidae						
18p*	*Acaeroplastes melanurus* (Budde-Lund, 1885)	MG887960	MG887945	MG887991	MG887912	Punta Menga (Sardinia)
2p*	*Agabiformius excavatus* Verhoeff, 1941	MG887955	—	MG888009	MG887921	Pafos (Cyprus)
3p		MG887956	—	—	MG887922	Pafos (Cyprus)
94cy	*Agabiformius lentus* (Budde-Lund, 1885)	ON219958	ON212535	—	—	Kourio (Cyprus)
20p*	*Brevurus masandaranus* Schmalfuss, 1986	—	—	MG888008	MG887919	Iran
13p*	*Caeroplastes porphyrivagus* (Verhoeff, 1918)	—	MG887932	MG887990	—	Toulon (France)
8p	*Leptotrichus kosswigi Strouhal, 1960*	MG887963	—	MG888013	MG887915	Pafos (Cyprus)
1KR		ON220034	ON212536	—	—	Kourio (Cyprus)
16p*	*Lucasius pallidus* Budde-Lund, 1885	—	—	ON312040	MG887917	Monte Arci (Sardinia)
17p*	*Mica tardus* (Budde-Lund, 1885)	MG887959	—	MG887996	—	Monte Coa Margine (Sardinia)
4p*	*Porcellio laevis* Latreille, 1804	MG887957	MG887936	MG887993	MG887913	Limassol (Cyprus)
5p*		MG887958	MG887937	MG887994	MG887914	Limassol (Cyprus)
10p*	*Porcellio nasutus* Strouhal, 1936	ON220051	—	—	—	Mt.Parnon (Greece)
10p*		MG887953	MG887944	MG887998	MG887910	Mt.Parnon (Greece)
11p*		MG887954	—	MG887999	MG887911	Mt.Parnon (Greece)
204z	*Porcellio* sp.	ON220052	ON212560	—	ON227372	Chrysoroyiatissa (Cyprus)
15z	*Porcellionides* sp.	—	—	—	ON227358	Larnaca (Cyprus)
18z		—	—	—	ON227359	Larnaca (Cyprus)
21p*	*Porcellionides cilicius* (Verhoeff, 1918)	—	—	—	ON227357	Agios Sozomenos (Cyprus)
21p *		—	—	—	MG887909	Agios Sozomenos (Cyprus)
19z	*Porcellionides pruinosus* (Brandt, 1833)	—	—	ON312052	ON227361	Limassol (Cyprus)
17z		—	—	ON312051	ON227360	Limassol (Cyprus)
17z		MG887950	MG887935	MG887989	MG887908	Larnaca (Cyprus)
1p*	*Proporcellio vulcanius* (Verhoeff, 1908)	MG887948	MG887933	MG887988	MG887906	Larnaca (Cyprus)
19p*	*Soteriscus laouensis* Taiti & Rossano, 2015	MG887964	MG887931	MG887997	MG887918	Oued Laou valley (Morocco)
15p*	*Thermocellio* sp.	MG887962	—	MG887995	—	Dar es salaam (Tanzania)
12p	*Tura sp.*	MG887966	MG887946	MG888001	MG887920	Mombasa (Kenya)
14p*	*Uramba triangulifera* Budde-Lund, 1910	MG887961	—	MG888002	MG887923	Aberdare (Kenya)
Scleropactidae						
26z	*Circoniscus bezzii* Arcangeli, 1931	—	—	—	—	Brazil
65T	*Kithironiscus paragamiani* Schmalfuss, 1995		ON212615	—	—	Cave Ag. Sofia Kythira (Greece)
Scyphacidae						
4z*	*Actaecia euchroa* Dana, 1853	—	—	MG888007	MG887930	New Zealand
10z	*Scyphax ornatus* Dana, 1853	ON219955	—	ON312058	—	New Zealand
Trachelipodidae						
24z*	*Levantoniscus bicostulatus* Cardoso, Taiti & Sfenthourakis, 2015	—	—	MN174811	MN234257	Pafos (Cyprus)
25z*		—	—	MG888000	MG887928	Pafos (Cyprus)
22z*	*Levantoniscus makrisi* Cardoso, Taiti & Sfenthourakis, 2015	—	ON212546	MN174810	MN234260	Pafos (Cyprus)
28cy	*Nagurus carinatus* (Dollfus, 1905)	ON220044	ON212541	ON312046	—	Cedar valley (Cyprus)
93cy		ON220045	ON212542	—	—	Kykkos monastery (Cyprus)
134z*	*Trachelipus aegaeus* (Verhoeff, 1907)	—	—	ON312073	MG887925	Naxos (Greece)
63T	*Trachelipus cavaticus* Schmalfuss, Paragamian & Sfenthourakis, 2004	ON220079	ON212614	—	—	Cave Landaka Chania, Crete (Greece)
144z*	*Trachelipus ratzeburgii* (Brandt, 1833)	—	—	MN174830	MN234279	Germany
64Tc	*Trachelipus* sp.	—	—	—	—	Cave Mougri Sisses, Crete (Greece)
?						
Exbr	*Exalloniscus brincki* Manicastri & Argano, 1986	ON219995	—	—	—	Sri Lanka
Synocheta						
Styloniscidae						
68T	*Cordioniscus* sp.	—	ON212524	—	—	Cave Spilia tis Agias at Katafygi (Peloponese)
27T		ON219981	—	—	—	Cave Dionysos Leonidion (Peloponnisos, Greece)
29T		ON219982	—	—	—	Cave Katofygi (Crete, Greece)
33Tc		—	ON212513	—	—	Cave of Sava Vardaki Stroumboulas (Greece)
38T		—	ON212491	—	—	Cave Katofygi, Crete (Greece)
42z*	*Styloniscus magellanicus* Dana, 1853	—	—	MN174832	—	Lago Argentino (Argentina)
Trichoniscidae						
145z	*Aegonethes cervinus* (Verhoeff, 1931)	ON219956	ON212562	—	ON227384	Dugopolje (Croatia)
146z		ON219957	ON212561	—	ON227385	Dračevac (Croatia)
61T	*Alistratia beroni* Andreev, 2004	ON219959	—	—	—	Cave Alistrati (Greece)
147z	*Alpioniscus balthasari* (Frankenberger, 1937)	ON219961	ON212551	ON312003	—	Kistanje (Croatia)
139z*	*Androniscus roseus* (C. Koch, 1838)	—	—	MN174824	MN234283	The Netherlands
201z	*Borutzkyella revasi* (Borutzky, 1973)	ON219968	ON212555	ON312011	—	Abkhazia (Caucasus)
202z		ON219969	ON212554	ON312012	—	Abkhazia (Caucasus)
149z	*Buddelundiella cataractae* Verhoeff, 1930	ON219971	—	—	ON227382	Šibenik (Croatia)
148z	*Buddelundiella* sp.	ON219972	—	—	—	Trogir (Croatia)
150z*	*Calconiscellus karawankianus* (Verhoeff, 1908)	ON219973	ON212556	MN174827	MN234277	Blato (Croatia)
196z	*Caucasocyphonethes cavaticus* Borutzky, 1948	ON219974	ON212572	ON312013	ON227377	Krasnodar (Caucasus)
197z		ON219975	ON212573	ON312014	ON227378	Krasnodar (Caucasus)
193z*	*Caucasocyphonethes* sp.	ON219977	ON212557	MN174825	MN234268	Ochamchira (Abkhazia)
194z*		ON219976	ON212558	MN174826	MN234269	Ochamchira (Abkhazia)
76Y	*Caucasonethes* sp.	ON219978	—	—	—	Ochamchira (Abkhazia)
200z	*Colchidoniscus kutaissianus* Borutzky, 1974	ON219980	—	ON312017	ON227380	Martvili (Georgia)
152z	*Cyphonethes herzegowinensis* (Verhoeff, 1900)	ON219984	ON212553	ON312020	—	Začir (Montenegro)
153z		ON219985		ON312021	—	Osojnik (Croatia)
154z	*Cyphopleon kratochvili* (Frankenberger, 1939)	ON219986	ON212574	—	—	Ključ (Bosnia and Herzegovina)
155z		ON219987	—	—	—	Ključ (Bosnia and Herzegovina)
160z	*Hyloniscus adonis* Verhoeff, 1927	ON220030	—	—	—	Sljeme (Croatia)
161z	*Hyloniscus* sp.	ON220031	ON212552	—	ON227381	Samobor (Croatia)
76T	*Kosswigius delattini* Verhoeff, 1941	—	ON212616	—	—	Thraki, Sapka (Greece)
132z	*Miktoniscus* sp.	—	—	ON312043	—	Clohars-fouesnant (France)
92z	*Miktoniscus patience* Vandel, 1946	—	—	ON312042	—	Madeira
164z	*Moserius percoi* Strouhal, 1940	ON220043	—	ON312045	—	Škocjan (Slovenia)
174z	*Tachysoniscus* cf. *austriacus* (Verhoeff, 1908)	ON220057	ON212550	ON312059	ON227391	Lovranska (Croatia)
59Y	*Tauronethes* cf. *lebedinskyi* Borutzky, 1949	—	—	—	—	Morcheka (Crimea)
60Y		—	ON212568	—	—	Morcheka (Crimea)
63Y		ON220074	—	—	—	Baidarskaya (Crimea)
185z*	*Tauronethes lebedinskyi* Borutzky, 1949	ON220075	—	MN174831	MN234272	Baidarskaya (Crimea)
186z		ON220076	ON212596	ON312072	—	Baidarskaya (Crimea)
61Y	*Tauronethes* sp.	ON220078	—	—	—	Bakhchsarai (Crimea)
62Y		ON220077	—	—	—	Sevastopol (Crimea)
176z	*Thaumatoniscellus speluncae* Karaman, Bedek, & Horvatović, 2009	—	—	—	ON227383	Skadanščina (Slovenia)
42T	*Trichonethes kosswigi* Strouhal, 1953	ON220080	ON212617	—	—	Cave Karami Archangelos (Rhodes, Greece)
66Y	*Trichoniscus aphonicus abchasicus* Borutzky, 1977	ON220081	ON212570	—	—	Sukhum (Abkhasia)
75Y		ON220083	ON212569	—	—	Sukhum (Abkhasia)
64Y	*Trichoniscus aphonicus codoricus* Borutzky, 1977	ON220084	ON212563	—	—	Ochamchira (Abkhasia)
65Y		—	ON212566	—	—	Ochamchira (Abkhasia)
68Y		—	ON212564	—	—	Ochamchira (Abkhasia)
69Y		ON220085	ON212580	—	—	Ochamchira (Abkhasia)
70Y		ON220086	ON212565	—	—	Ochamchira (Abkhasia)
47T	*Trichoniscus cavernicola* Vandel, 1958	ON220087	ON212522	—	—	Pathole Xepatomeni Latsiola, Crete (Greece)
71Y	*Trichoniscus* cf. *aphonicus abchasicus* Borutzky, 1977	ON220088	—	—	—	Sukhum (Abkhasia)
72Y		ON220082	—	—	—	Gudauta (Abkhasia)
67Y	*Trichoniscus* cf. *aphonicus codoricus* Borutzky, 1977	ON220089	ON212567	—	—	Ochamchira (Abkhasia)
73Y	*Trichoniscus* cf. *gudauticus* Borutzky, 1977	ON220090	ON212571	—	—	Gudauta (Abkhasia)
59T	*Trichoniscus fragilis* Racovitza, 1908	ON220091	ON212521	—	—	Varathro Stenou Logiou, Crete (Greece)
1T	*Trichoniscus intermedius* Vandel, 1958	ON220092		—	—	Pothole Xylouri Tafkos, Crete (Greece)
2T	*Trichoniscus lindbergi* Vandel, 1958	ON220095	ON212523	—	—	Pathhole tafkos xepatomenos, Crete (Greece)
20T		ON220093	ON212613	—	—	Pathole Dalamoutou, Crete (Greece)
25T		ON220094	—	—	—	Pathole Tafkos Myristis, Crete (Greece)
4T		ON220096	ON212612	—	—	Cave Geranion, Crete (Greece)
5T		ON220097	—	—	—	Cave Kamilari Tylisos, Crete (Greece)
23z*	*Trichoniscus provisorius* Racovitza, 1908	—	—	MN174834	MN234259	Aphrotide's baths (Cyprus)
33z*		—	—	MN174836	MN234253	Pitargou (Cyprus)
21T	*Trichoniscus pusillus* Brandt, 1833	ON220098	—	—	—	Cave Peristeri (Peloponese)
8T		ON220099	—	—	—	Cave Perama (Greece)
9T	*Trichoniscus* sp.	ON220102	—	—	—	Pathole Votsos
24T		ON220100	—	—	—	Pothole Megalos Votsos (Greece)
6T		ON220101	—	—	—	Pathhole Maratholakou Liliano (Greece)
107cy*		—	ON212549	MN174835	MN234286	Afrodite's baths (Pafos)
74Y		ON220103	—	—	—	Gulripsh (Abkhasia)
177z	*Troglocyphoniscus absoloni* Strouhal, 1939	ON220104	ON212576	—	—	Lumbarda (Croatia)
178z		ON220105	ON212575	ON312074	ON227379	Blato (Croatia)
Microcheta						
Mesoniscidae						
6z*	*Mesoniscus alpicola* (Heller, 1858)	ON220042	—	MN174829	MN234249	Lombardia (Italy)
Tylidae						
Tylidae						
133z*	*Helleria brevicornis* Ebner, 1868	—	ON212510	MN174843	MN234285	Croix-Valmer (France)
138z	*Tylos ponticus* Grebnicki, 1874	—	—	ON312075	ON227388	Konnos (Cyprus)
37cy		—	—	ON312078	ON227386	Governor's Beach (Cyprus)
59cy		—	—	ON312079	ON227387	Konnos (Cyprus)
210z		—	—	ON312076	ON227389	Cape Greco (Cyprus)
211z		—	—	ON312077	ON227390	Cape Greco (Cyprus)
212z*		—	—	MN174844	MN234265	Cape Greco (Cyprus)
Ligidiidae						
Ligidiidae						
51Y	*Ligidium cavaticum* Borutzky, 1950	ON220035	—	—	—	Krasnodar (Caucasus)
53Y		ON220036	—	—	—	Krasnodar (Caucasus)
54Y		ON220037	ON212611	—	—	Krasnodar (Caucasus)
57Y		ON220038	—	—	—	Krasnodar (Caucasus)
58Y		ON220039	—	—	—	Krasnodar (Caucasus)
59Y		ON220040	—	—	—	Morcheka Mt. (Crimea)
136z	*Ligidium ghigii* Arcangeli, 1928	—	—	MN174818	MN234284	Naxos (Greece)
Ligiidae						
61cy*	*Ligia italica* Fabricius, 1798	—	—	ON312038	MN234250	Konnos (Cyprus)
188z	*Tauroligidium* cf. *stygium* Borutzky, 1950	—	—	—	—	Baidarskaya (Crimea)
189z*		—	—	MN174819	MN234271	Baidarskaya (Crimea)
190z*		—	—	MN174820	MN234270	Baidarskaya (Crimea)
191z		—	—	—	—	Bakhchsarai (Crimea)
21Y		ON220058	ON212598	ON312060	—	Bakhchsarai (Crimea)
22Y		ON220059	ON212604	—	—	Bakhchsarai (Crimea)
23Y		ON220060	—	ON312061	—	Bakhchsarai (Crimea)
24Y		—	ON212603	ON312062	—	Baidarskaya (Crimea)
26Y		ON220061	—	ON312063	—	Baidarskaya (Crimea)
27Y*		ON220062	ON212610	—	MN234256	Bakhchsarai (Crimea)
29Y		ON220063	ON212607	ON312064	—	Baidarskaya (Crimea)
30Y*		ON220064	ON212597	MN174821	MN234255	Bakhchsarai (Crimea)
31Y		ON220065	—	ON312065	—	Bakhchsarai (Crimea)
32Y		ON220066	ON212609	ON312066	—	Bakhchsarai (Crimea)
36Y		ON220067	ON212606	ON312067	—	Baidarskaya (Crimea)
37Y		ON220068	ON212608	ON312068	—	Baidarskaya (Crimea)
38Y		ON220069	ON212605	ON312069	—	Morcheka (Crimea)
40Y		—	ON212594	—	—	Morcheka (Crimea)
42Y		ON220070	—	—	—	Bakhchsarai (Crimea)
44Y		ON220071	—	—	—	Baidarskaya (Crimea)
198z	*Tauroligidium stygium* Borutzky, 1950	ON220072	ON212591	ON312070	—	Baidarskaya (Crimea)
199z		ON220073	—	ON312071	—	Baidarskaya (Crimea)
39Y	*Typhloligidium* cf. *karabijajlae* Borutzky, 1962	—	ON212592	—	—	Belogorsk (Crimea)
48Y		—	ON212585	—	—	Belogorsk (Crimea)
192z	*Typhloligidium coecum* (Carl, 1904)	ON220108	ON212588	—	—	Simferopol (Crimea)
20Y		ON220109	ON212595	ON312080	—	Simferopol (Crimea)
28Y		—	ON212586	—	—	Simferopol (Crimea)
33Y		ON220110	ON212589	ON312081	—	Simferopol (Crimea)
35Y		ON220111	ON212584	ON312082	—	Simferopol (Crimea)
45Y*		ON220112	ON212587	MN174822	—	Simferopol (Crimea)
25Y	*Typhloligidium karabijajlae* Borutzky, 1962	ON220113	ON212601	ON312083	—	Belogorsk (Crimea)
34Y		ON220114	ON212602	ON312084	—	Belogorsk (Crimea)
41Y		ON220115	ON212590	—	—	Belogorsk (Crimea)
43Y		ON220116	—	—	—	Belogorsk (Crimea)
195z	*Typhloligidium kovali* Gongalsky & Taiti, 2014	ON220117	ON212581	ON312085	—	Kabardino-Balkaria (Caucasus)
47Y		ON220118	ON212579	—	—	Kabardino-Balkaria (Caucasus)
19Y	*Typhloligidium lithophagum* Turbanov & Gongalsky, 2016	ON220119	ON212582	ON312086	—	Simferopol (Crimea)
46Y		ON220120	ON212583	—	—	Simferopol (Crimea)
50Y*	*Typhloligidium* sp.	ON220121	ON212600	MN174823	MN234251	Martvili (Georgia)
56Y		ON220122	—	—	—	Martvili (Georgia)
49Y		—	ON212599	—	—	Martvili (Georgia)
Asellota						
Asellidae						
209z*	*Asellus aquaticus* (Linnaeus, 1758)	—	—	MN174846	MN234267	Kozani (Greece)
Valvifera						
Idoteidae						
213z*	*Idotea chelipes* (Pallas, 1766)	—	—	MN174840	MN234263	Ansedonia (Italy)
214z*		—	—	MN174839	MN234264	Ansedonia (Italy)
Sphaeromatidea						
Sphaeromatidae						
215z*	*Sphaeroma serratum* (Fabricius, 1787)	—	—	MN174842	MN234262	Ansedonia (Italy)
216z*		—	—	MN174841	MN234261	Ansedonia (Italy)

## Conclusion

Taking advantage of the universal and cost-effective nature of the proposed protocol a new potential for the production of genetic data could be foreseen. Based on this protocol retrieved data could be included in multiple datasets given that the same gene regions are targeted. Beyond the data published within the framework of this study, scientific research in related fields such as Isopod systematics, evolution, phylogeny e.t.c. could be facilitated by the application of the proposed protocol. It is worth noting that the majority of available specimens were collected more than two decades ago and a considerable number of them were ill-preserved for a long time (i.e., in 70% alcohol or formaldehyde). Hence, the absence of data in some cases might be attributed to the bad quality of extracted DNA or the lack of effort since we did not aim to sequence all loci for all specimens.

## Data availability

All data were deposited in NCBI Genbank. Accession numbers are provided in the main text.

## Declaration of Competing Interest

The authors declare that they have no known competing financial interests or personal relationships that could have appeared to influence the work reported in this paper.

## Data Availability

All data were deposited in NCBI Genbank. Accession numbers are provided in the main text.
